# Farmland biodiversity monitoring through citizen science: A review of existing approaches and future opportunities

**DOI:** 10.1007/s13280-023-01929-x

**Published:** 2023-11-16

**Authors:** Andy Ruck, René van der Wal, Amelia S. C. Hood, Alice L. Mauchline, Simon G. Potts, Michiel F. WallisDeVries, Erik Öckinger

**Affiliations:** 1 Department of Ecology, Swedish University of Agricultural Sciences, Box 7044, 75007 Uppsala, Sweden; 2 School of Agriculture, Policy and Development, Centre for Agri-Environmental Research, University of Reading, Reading, RG6 6EU UK; 3 De Vlinderstichting/Dutch Butterfly Conservation, P.O. Box 506, 6700AM Wageningen, The Netherlands

**Keywords:** Agricultural landscapes, Biodiversity monitoring, Citizen science, Farmer engagement, Review, Typology

## Abstract

Biodiversity monitoring in agricultural landscapes is important for assessing the effects of both land use change and activities that influence farmland biodiversity. Despite a considerable increase in citizen science approaches to biodiversity monitoring in recent decades, their potential in farmland-specific contexts has not been systematically examined. This paper therefore provides a comprehensive review of existing citizen science approaches involving biodiversity monitoring on farmland. Using three complementary methods, we identify a range of programmes at least partially covering farmland. From these, we develop a typology of eight programme types, reflecting distinctions in types of data collected and nature of volunteer involvement, and highlight their respective strengths and limitations. While all eight types can make substantial contributions to farmland biodiversity monitoring, there is considerable scope for their further development—particularly through increased engagement of farmers, for whom receiving feedback on the effects of their own practices could help facilitate adaptive management.

**Supplementary Information:**

The online version contains supplementary material available at 10.1007/s13280-023-01929-x.

## Introduction

Biodiversity loss is a major global issue stemming from “unprecedented, unsustainable and growing impacts on wild species from human activities” (IUCN 2019, p. 1). Loss of biodiversity in agricultural landscapes is a primary contributor to this (FAO 2019), with more than one third of the global land area under agricultural use (FAO 2020)*.* Biodiversity within agricultural landscapes is particularly threatened by the expansion and intensification of agriculture, and subsequent loss of (semi-) natural habitats that support certain species (Lanz et al. 2018; Rigal et al. 2023). Additionally, abandonment of marginal farmland areas is resulting in the loss of species specifically adapted to farmland habitats (Isbell et al. 2019).

In recent decades, many actions have been introduced or encouraged to promote biodiversity in agricultural areas across the world—for example, through agri-environmental schemes in the USA (Biffi et al. 2021), Africa (Lawin and Tamini 2019) and Australia (Ansell et al. 2016), as well as measures embedded in the European Union’s Common Agricultural Policy (Leventon et al. 2017). Monitoring the effects of such actions is necessary for assessing the extent to which they have the intended positive effects on biodiversity, and whether they are sufficient to halt biodiversity loss (Kleijn and Sutherland 2003). Furthermore, as well as more widely improving species distribution and abundance, there is increasing evidence that restoring, enhancing or conserving farmland biodiversity can in fact have positive impacts on agricultural productivity (e.g. Bommarco et al 2013; Pywell et al. 2015), and monitoring can also be a means of gathering further evidence of such benefits. Thus, there is a clear and ongoing need for farmland-specific biodiversity data, and by extension, people to collect these data.

As an approach to monitoring biodiversity in general, ‘citizen science’ initiatives have increased hugely over the past 20 years (Pocock et al. 2017; Thornhill et al. 2021). ‘Citizen science’ broadly refers to the voluntary contribution of members of the public to scientific research (Dickinson and Bonney 2012), with biodiversity being a major area of focus within this. The term, however, represents a broad spectrum of approaches (Haklay et al. 2021) and epistemologies (Kasperowski and Kullenberg 2019) that is by no means restricted to biodiversity. Even within biodiversity-focused citizen science, programmes have a variety of purposes, from monitoring national and even international-scale trends (Stuber et al. 2022), to assessing the effects of local actions to promote biodiversity (Griffiths-Lee et al. 2022). From a data collection perspective, especially with the rise of internet-based platforms, biodiversity informatics infrastructures (Peterson et al. 2023) and the use of mobile applications (Dehnen-Schmutz et al. 2016), citizen science has enabled the collection of volumes of data that were previously impossible (Chandler et al. 2017). It can also reduce costs compared to monitoring by professional biologists (Lasky et al. 2021), and make use of the species-specific expertise of a great many knowledgeable amateurs (Viola et al. 2022).

A considerable body of research now also demonstrates the potential of citizen science as a means of engaging new audiences with science and biodiversity (Bonney et al. 2009), and in turn, contributing towards conservation, environmental and wider societal goals. On an individual level, for example, participation in citizen science projects can increase scientific literacy (Cronje et al. 2011), raise awareness of environmental and conservation issues (Goudeseune et al. 2020) and lead to or at least reinforce positive attitudes towards conservation behaviours (Toomey and Domroese 2013). On a societal level, Strasser et al. (2019) point to a process of “‘democratizing’ science” (p. 66) that citizen science can contribute to—one that challenges the separation between the production of knowledge by professional scientists in dedicated research institutions and the public as “consumers” of such knowledge (p. 53). As well as increasing public trust in science (Goudeseune et al. 2020), such a ‘democratisation’ can ideally lead to greater distribution of knowledge, and a society in which citizens and citizen groups are more able to participate in evidence-based policy development and decision-making (Kasperowski and Kullenberg 2019). Considering these potential benefits to science, participants and society, it is clear that both the collection of increased volumes of data, and the engagement of farmers and other members of agricultural communities with biodiversity monitoring, can potentially act as a means to the same end: the conservation, restoration, or enhancement of biodiversity.

When applied to farmland specifically, citizen science appears to have considerable potential, both in terms of data collection and the engagement of farmers and other members of agricultural communities (hereafter ‘farmers’). From a data collection perspective, citizen science approaches can be used to monitor wider trends in farmland biodiversity, identify threats to farmland biodiversity and assess specific agricultural practices aiming to promote biodiversity as highlighted above. Furthermore, farmers in particular are well-placed to participate in such monitoring, given their proximity to and daily engagement with what would be their data collection sites. These two factors may also help to attract farmers to citizen science on farmland, although many of the identified intrinsic and extrinsic motivations of those contributing to citizen science are of a different nature (West et al. 2021). In terms of engagement, farmers are also a particularly important audience, given that they usually have at least some degree of agency to adapt their farming practices as a direct response to the results of data collection on their own land (Billaud et al. 2021). Additionally, their (potentially) increased scientific literacy and trust in science might help to counter the influence of other prominent actors, such as fertiliser and machinery suppliers, in farmers’ decision-making processes (Kleijn et al. 2019).

Despite this potential, however, the broader picture of how citizen science has been applied to farmland biodiversity monitoring—the overall extent, the approaches taken, the types of data collected and for what purposes these can be used—remains unclear. While previous reviews have highlighted trends in broader categories of citizen science, to our knowledge there has been no comprehensive review focusing on farmland biodiversity monitoring specifically. Pocock et al. (2017, p. 1) provide a review of “ecological and environmental citizen science” programmes generally, showing how these have evolved and become more diverse. Others have reviewed citizen science initiatives on farmland, but with a focus on—for example—testing new technologies and crop varieties (Van der Gevel et al. 2020), rather than biodiversity. Another review by Herzog and Franklin (2016) focuses on attributes and design choices of eleven large-scale programmes for farmland biodiversity monitoring, including several that are citizen science-based, but does not focus specifically on the role of citizen scientists.

Here, we build upon these studies with a comprehensive review of the different types of citizen science approaches to biodiversity monitoring on farmland, and set out a typology of eight programme types. We then discuss the overall potential of citizen science for farmland biodiversity monitoring and the ways in which these could be expanded, and the key advantages and limitations of the different programme types in terms of both data collection and volunteer (especially farmer) engagement.

## Materials and methods

Through this review, we sought to identify citizen science initiatives that are, or have been, used to monitor biodiversity on farmland, and to develop a typology based on these. Importantly, our goal was not to identify every citizen science programme ever employed on farmland. Rather, we aimed to conduct a wide-ranging review of the different *types* of approaches, and what these can offer to farmland biodiversity monitoring. The scope of our searches was global, although limited to information provided in English, Swedish and Dutch, and somewhat weighted towards the countries in which co-authors are based (see “Partner contributions” below). This section details the various stages of the process followed.

### Three searches

In identifying programmes, we used three complementary methods that, taken together, provided an effective combination of systematic and illustrative review methods. Searches were conducted initially between June and August 2021, and updated in September 2022, in the following order.

- **(i) Literature search.** We conducted an extensive search of the Web of Science Core Collection. Here, we first employed a narrow ‘title search’ that we felt would quickly identify the most relevant articles. Second, to broaden the range of relevant papers, we performed an ‘author keyword search’—that is, a search of keywords provided by authors—using a wider set of terms. This accounted for the relatively recent emergence of the term ‘citizen science’, and attempted to cover the numerous related terms with a similar meaning (e.g. ‘public science’, ‘community science’). We also combined these terms with specific taxa that may have been referred to instead of the generic ‘biodiversity’, focusing on taxa that are commonly used as indicators for farmland biodiversity. Full lists of the search terms used, and papers identified, are included in the supplementary materials (Appendices S1 and S2).
- **(ii) Google search**. We then conducted a further search using Google to identify any clearly relevant schemes overlooked in our literature search (see Appendix S1 for search terms). This search was conducted using Google in ‘incognito’ mode and with all ‘cookies’ removed to avoid the results being influenced by previous search histories. We screened the first 100 results from each search. While our Web of Science search provided a wide-ranging review of scientific literature that was global in scope, we knew that not all monitoring programmes were likely to be referred to in scientific papers. This Google search, then, enabled us to fill these gaps with programmes that had not yet been mentioned in the scientific literature. We chose to focus on farmland-specific programmes here because we predicted that a wider Google search (e.g. for ‘citizen science’ generally) would likely identify too many programmes, and that it would be difficult to determine whether or not these included farmland.
- **(iii) Partner contributions:** Following the searches described above, experts in the UK, Sweden and the Netherlands (all co-authors in this paper) then used their existing knowledge and conducted ad-hoc searches and consultations to identify additional programmes. These contributions enabled us to draw upon the context-specific knowledge of experts in three national contexts in which citizen science is well-established (most additional programmes identified were based in partners’ home countries, with a few exceptions), to identify further relevant programmes that had not (yet) been referred to in the academic literature. With the Web of Science and Google searches both limited to the English language, it also expanded the review to include programmes on which information was provided only in Swedish and Dutch.

### Inclusion criteria

We had three clear criteria for identifying programmes that ran through each of the three searches. First, programmes must take place on *farmland,* although not necessarily exclusively. That is, farmland could be just one of a number of landscape or habitat types included in the programme, but it must either make some reference to including farmland among the habitat types covered (for example, on their website), or be known to include land used for commercial food production (e.g. arable, pastoral, agroforestry) by project partners. Rather than starting with a particular definition of ‘farmland’ here, we simply included programmes where the habitats in which monitoring took place were defined as such in academic articles or by project partners. To ensure a breadth of potential definitions, our Web of Science author keyword search used the terms: farm*OR agri*OR agro* (see Appendix S1). Such interpretations of ‘farmland’ or ‘agricultural land’ may not account for the full range of habitats that could in some contexts be considered agricultural, especially given that the global coverage of citizen science as a whole is heavily concentrated in Europe and North America (Chandler et al. 2017). Second, the programme must focus on *biodiversity* or components thereof; and thirdly, the programme must involve monitoring carried out by *non-professionals* (citizen scientists). We only included programmes that met all three of these criteria. This led to the exclusion of many programmes that met only one or two of these criteria—such as those where farmers had participated in monitoring of something other than biodiversity (e.g. the effects of new agricultural technologies). We included programmes that were discontinued as well as active.

### Programme coding

Programmes were coded into a spreadsheet that reflected two key themes: first, the type of data collected and what these can offer to farmland biodiversity monitoring, and second, the ways in which volunteers, especially farmers, are engaged in a given programme. The themes of data type and volunteer engagement were reflected in 20 columns in our spreadsheet, which included the programme location, aims, scale, organising bodies, species or taxa monitored, monitoring methods, number of sites covered, frequency of data collection, programme strengths and weaknesses, and the stages of the scientific process in which volunteers were involved (see Appendix S3).

### Analysis and development of the typology

The programme details provided in the database then formed the basis of our analysis and the development of our typology. The typology was developed collectively by authors 1, 2 and 7, and refined with feedback from the remaining authors. Our analysis of the identified programmes was based on our familiarity with the wider citizen science literature, and practical knowledge and experience of citizen science programmes. The identification of the programme types comprising our typology, then, can be considered a situated interpretive process (Clarke et al. 2017) that combined some prior sense of the distinctions that *might* form a typology, with a careful analysis of the identified programmes.

For defining the nature of volunteer participation, we use Bonney et al.’s (2009) typology of ‘contributory’, ‘collaborative’, and ‘co-created’ projects. In ‘contributory’ projects, volunteers’ contributions are limited to the collection and submission of data to a project designed by researchers or conservation professionals. ‘Collaborative’ projects are still principally designed by scientists, but may also involve volunteers in (for example) refining the project design, or disseminating findings. Finally, in a ‘co-created’ model, volunteers work together with scientists from the very beginning of the research process, identifying the question or issue to be addressed, and then remaining involved in all stages of the scientific process. We refer to this scale of participation throughout our “Typology” and “Discussion and conclusions” sections.

### Note on identification of ‘general’ programmes

While not intended to be exhaustive, the identification of ‘farmland-specific’ programmes identified in the typology can be considered a comprehensive search complemented by examples drawn from the context-specific knowledge of partners. The identification of ‘general’ programmes (Types 1 to 3, see Table 1), meanwhile, can be considered more illustrative of a general trend than in any way comprehensive, for two main reasons: the large number of these programmes identified through partner contributions, and the fact that these contributions were heavily weighted towards the countries in which those partners are based. We decided that there was little to be learned from continuing to add programmes to the database that included farmland to a limited extent, but where this was not a strong focus. We can therefore be certain that there are many more ‘general’ programmes that partly take place on farmland than those included in this review, particularly in countries other than the UK, Sweden and the Netherlands.

**Table 1:** Summary of key features of the three ‘general’ programme types identified in this review, and how they were identified

	Type 1: general; free method and site selection	Type 2: general; fixed method, free site selection	Type 3: general; fixed method and site selection
Scale of coordination	National/international	National	National
Data collection aim	Collect large–scale data on species distributions	Monitor general large-scale biodiversity trends	Monitor general large-scale biodiversity trends
Site selection	Free (by volunteers)	Free (by volunteers)	Fixed (systematic on national scale)
Methods	Free	Fixed	Fixed
Open-ended or time-limited	Open-ended	Open-ended	Open-ended
Scale/type of data available	Large-scale longitudinal data providing records of species present	Large-scale longitudinal data on general trends	Large-scale longitudinal data on general trends (possible to systematically account for farmland)
Indicative number of sites (max. per annum)	100 000's	10 000’s	1000's
Volunteer involvement	Contributory: monitoring/ submission of data	Contributory: monitoring/submission of data	Contributory: monitoring/submission of data
*Identified through*			
Lit search	0	0	13
Google search	1	1	0
Experts	25	16	8
Number of programmes	**26**	**17**	**21**

## Results and typology overview

We identified 106 programmes that met our search criteria (see Appendix S4 for a full list of these). Our literature search returned 326 papers, which we screened to include 27 programmes. Our Google search identified 11 additional programmes and our partner contributions added a further 68 programmes. There was considerable geographical spread across the programmes identified, with twenty different countries represented. The vast majority, however, were located in Europe—a global hotspot for citizen science as a whole (Requier et al. 2020). This was the case also when only considering the programmes identified through the literature search, in which 19 out of 24 were European. There were just five exceptions to this, albeit notable ones, located in the USA (×2), New Zealand, South Africa, and Taiwan (see Type 6 examples in Typology). Across all three search methods (literature search, Google search, partner contributions) we identified 95 European and 11 non-European programmes. The dominant data collection methods across all programme types were some form of point, route or transect count, although there was great variation in the exact types of count used—from annual counts of a small patch (Monitoring of Danish Orchids), to fixed routes along roads running through farmland areas (Coordinated Avifaunal Road Counts, South Africa).

Although a significant number of programmes are specifically targeted at farmers and other members of agricultural communities, our review suggests relatively little involvement of this audience across our sample as a whole. That is, data collection in the identified programmes is often carried out by volunteers from outside of this community, and where this *is* done by farmers, in most cases their involvement does not extend to other stages of the scientific process (e.g. Josefsson et al. 2017; Billaud et al. 2021).

## Typology

From the identified programmes, we developed a typology of eight programme types, as outlined in this section. The first six programme types are based on the type of data collected, and represent three further distinctions within this: first, whether the programme focuses only partially or entirely on farmland (‘general’ or ‘farmland-specific’); second, whether there are specific protocols to follow when collecting data, or volunteers are invited to simply submit observations at any given time or place (‘fixed’ or ‘free’ method), and; third, whether monitoring sites are chosen by volunteers or selected more systematically by researchers or a coordinating organisation (‘free’ or ‘fixed’ site selection). The final two programme types (Types 7 and 8) represent a small number of programmes that stand out for the notable ways in which they engage farmers, rather than for the data collection they enable. Tables 1 and 2 summarise the eight programme types making up this typology—first ‘general’ (Table 1) and then ‘farmland-specific’ (Table 2) programmes.

**Table 2:** Summary of the key features of the five ‘farmland-specific’ programme types identified in this review, and how they were identified

	Type 4: farmland-specific; free method and site selection	Type 5: farmland-specific; fixed method, free site selection	Type 6: farmland-specific; fixed method and site selection	Type 7:upporting small-scale farmer-led investigations	Type 8: farmer engagement
Scale of coordination	National	National	Local, habitat or practice-specific	Locally-specific	National
Data collection aim	Collect large-scale data on farmland species	Monitor large-scale trends on farmland	Measure effects of specific habitats/ Practices	Answering specific (local) questions	NA (farmer engagement)
Site selection	Free (by volunteers)	Free (by volunteers)	Fixed (according to specific criteria)	Fixed (according to specific criteria)	Free (by volunteers)
Methods	Free	Fixed	Fixed	Fixed	Free
Open-ended or time-limited	Open-ended	Open-ended	Time-limited	Time-limited	NA
Scale/type of data available	(Potentially) Large-scale data on species present on farmland	Large-scale, general trends on farmland	Measuring effects of specific practices on farmland biodiversity	Measuring effects of specific practices (not only on biodiversity)	NA
Indicative number of sites (max. per annum)	Unclear	1000's	100’s	4–15	Unclear
Farmer/volunteer involvement	Contributory: monitoring/submission of data	Contributory: monitoring/submission of data	Contributory: monitoring/submission of data	Co-created: throughout research process	Monitoring
Identified through:					
Lit search	0	2	9	0	0
Google search	1	10	2	0	4
Experts	0	5	7	2	0
Number of programmes	1	17	18	2	4

Type 1: general; free method and site selection Programmes of this type consist of large-scale initiatives that encourage members of the public to submit records of sightings at any time or place, usually through a website or smartphone app. These include platforms such as iNaturalist (worldwide) and iSpot (UK/Ireland/Southern Africa), where members of the public are encouraged to submit records of sightings of any species, in any location or habitat type, and are helped to identify it through online identification tools and a knowledgeable online community. There are also taxon-specific platforms such as eBird (USA), and some focused on less commonly monitored taxa such as reptiles (RecordPool, UK).

Common to all programmes of this type is a lack of a standardised method or specified time commitment—volunteers can submit records as often or infrequently as they choose (though full species lists and provision of observation duration may be encouraged as is the case in eBird, for example). There is also no stated requirement, in any of these programmes, for volunteers to have any particular expertise. In all programmes, volunteers’ participation is limited to submitting observations or results—that is, they have no involvement in, for example, designing the study or analysing data.

**Type 1 example: Naturens Kalender (Sweden)**

Similar to Nature's Calendar in the UK, this programme aims to monitor changes in phenology, especially during spring. There are several separate ‘calendars’, or recording categories, such as for birds, flowers, fungi and insects; and within each, volunteers can select from a long list of species to record which they have seen. Anyone can record sightings, which then appear on an interactive map visible to all users. Recordings might include the first wood anemones flowering in spring, or the first calling barn owls of the year. With this focus on phenology of common species, over time Naturens Kalender will help to build up a picture of the effects of climate change on those species. The programme is run by Swedish Phenology Network—a network of numerous universities, government agencies, and wildlife organisations.

Type 2: general; fixed method, free site selection In Type 2 programmes, volunteers are required to follow a fixed method, but similarly to Type 1 programmes, monitoring sites are chosen by volunteers, rather than being systematically selected. Typically, volunteers are asked to visit the same site multiple times—for example, carrying out a regular timed count of pollinators visiting a particular flower patch (e.g. UK pollinator monitoring scheme—FIT counts), or returning to a chosen site repeatedly over the season and in subsequent years, as is the standard method in several national schemes forming part of the European Butterfly Monitoring Scheme (Van Swaay et al. 2008). Also unlike the previous type, these programmes all focus on a particular taxonomic or functional group of species—for example, pollinators, common birds, or vascular plants. Again, volunteers’ participation is limited to submitting observations or results, and these programmes can therefore be considered typical of the ‘contributory’ model of volunteer engagement (Bonney et al. 2009).

**Type 2 example: X:Polli-Nation timed counts (United Kingdom)**

This pollinator-focused programme offers two ways for volunteers to participate, one of which consists of carrying out a standardised timed survey of small plots chosen by the volunteers (the other is a ‘Type 1’ scheme based on photo submission from any time or place). These ‘timed counts’ are a continuation of the citizen science element of the school-based Polli:Nation project, which took place between 2016 and 2018 in over 200 UK school grounds. The project is coordinated by researchers from the UK’s Open University, and our UK-based co-authors report that farmland is relatively well-represented within it. Furthermore, the well-developed survey and interpretation materials have the potential to be used in a farming context. The programme generates large-scale species distribution data, but its wider aims are to engage and train people to recognise species and to change how they think about and care for local green spaces.

Type 3: general; fixed method and site selection Type 3 are predominantly large-scale initiatives aiming to gather general, usually national-scale data on particular taxa or species, and are therefore also not specifically focused on farmland. Most of the programmes identified are national bird monitoring schemes. The key difference from Types 1 and 2 is that instead of relying on the opportunistic submission of records, sites are pre-selected by the coordinating organisation and allocated to volunteers. In the British Trust for Ornithology’s (BTO) Breeding Bird Survey (UK), for example, transects are selected using a form of stratified random sampling from the country’s national grid. Other monitoring schemes employing a systematic selection of sites include the Catalan Butterfly Monitoring Scheme and the UK Pollinator Monitoring Scheme.

These programmes require volunteers to make repeat visits to the same allocated site to complete their counts. While regular, however, the required commitment tends not to be frequent. It is common that counts are carried out annually, while other programmes require (at most) three or four surveys during a summer season. These programmes, as with Types 1 and 2, employ a typical ‘contributory’ model—that is, they are designed by researchers or conservation professionals, and volunteers’ contributions are limited to the collection and submission of data.

Compared to Type 1, there is also more emphasis on volunteers in Types 2 and 3 having at least some existing expertise—in particular species identification skills, which are necessary for ensuring the accuracy of data collected across a representative selection of sites. The BTO Breeding Bird Survey (UK) appears typical of programmes of this type, stating that “you don’t need to be an expert to take part, but you need to be able to identify the common birds you are likely to encounter… by sight and sound” (British Trust for Ornithology, n.d). BTO also runs training courses on birdwatching and produce high quality ‘differences between similar species’ videos, although these do not specifically prepare volunteers for contributing to this programme.

**Type 3 example: Suivi Temporel des Oiseaux Communs (France)**

*Suivi Temporel des Oiseaux Communs* is France’s national breeding bird survey, and a typical example of a Type 3 programme. Volunteers annually carry out ten counts at fixed points within a square selected by the organisations coordinating the project – a partnership between the French Natural History Museum, the NGO Ligue pour la Protection des Oiseuax (LBO), and the government agency Office Français de la Biodiversité. National bird monitoring programmes typically employ the same methods, following guidelines set out by the Pan-European Common Bird Monitoring Scheme. They are also typically long-running, with this survey dating back to 1989.

Type 4: farmland-specific; free method and site selection The first of five ‘farmland-specific’ programme types, this type consists of just one programme identified in our review—the UK-based Rare Arable Flowers App. As shown by the larger number of programmes in Type 1, most programmes inviting volunteers to submit opportunistic observations at any time or place do not focus on a particular landscape type, and thus appear in the ‘general’ category. All ‘farmland-specific’ programmes in Types 5–7, meanwhile, employ some form of fixed method and required time commitment. This app, however, is notable for its focus on gathering purely opportunistic data on farmland-specific species, as well as on farmers as a clear target audience.

**Type 4 example: Rare Arable Flowers App (United Kingdom)**

As with the platforms categorised under Type 1, this app encourages users to submit sightings at any time or place. It is, however, unique in its specific focus on wildflowers typically found on arable land, and includes a photographic guide to 121 featured species. The app was set up by the UK’s Biological Records Centre (BRC), which is supported by the research institute UK Centre for Ecology & Hydrology (UKCEH), in collaboration with Plantlife, Natural England and the Botanical Society of Britain and Ireland. There is a clear targeting of farmers as a key user group. The introduction to the app on the BRC website describes arable wildflowers as “an important part of our cultural heritage” and includes examples of the potential ecosystem services provided by them. There is also information given about each featured species, including suggestions for the “most suitable conservation management options“, and the app is mentioned in several articles in the industry magazine/website Farmer’s Weekly. Ultimately, through the app, the BRC aims to gather large-scale distribution data on arable wildflowers to “inform efforts for their conservation”.

Type 5: farmland-specific; fixed method, free site selection These programmes consist of large-scale initiatives that focus specifically on farmland, but where participants select their own sites on which to monitor. Examples include farmland bird monitoring schemes (e.g. GWCT Partridge Count Scheme, UK), surveys of arable weeds (Inventera Åkerogräs, Sweden), protocols for counting bees, earthworms and butterflies that feed into a national recording scheme (Observatoire Agricole de la Biodiversité, France), and surveys of birds nesting in specially-installed nest boxes (Songbird Farm Trail, USA). In all programmes of this type, while the selection of sites is ‘free’, a fixed method of recording is used.

**Type 5 example: Big Farmland Bird Count (United Kingdom)**

This programme is a national bird monitoring scheme with a specific focus on farmland and the engagement of farmers, and where monitoring sites are selected by participants (usually an area of their own farm). Since it began in 2014, participation has expanded so that over 1800 farmers took part in 2021, between them recording over 130 species across 2.5 million acres. Participants undertake one count during winter (between the 5th and 21st February). They are asked to spend around 30 minutes recording the number of birds for each species present on one particular area of a farm, as well as provide information on the types of habitat and cropping on and adjacent to the count site. The focus on farmer engagement is clear from the advice that “counting should take place at first light as this is when the birds are most active. However, it is more important that you take part, so timings should suit you”. The programme is run by the Game and Wildlife Conservation Trust (GWCT), an NGO that also runs a more species-specific monitoring programme: the Partridge Count Scheme.

These programmes are a mixture in terms of the type of volunteers they aim to engage. Many are clearly targeted at farmers to engage them with the biodiversity on their land. For example, the Game and Wildlife Conservation Trust (GWCT) reports that “thousands of farmers, gamekeepers and land managers around the UK are setting off across their fields to count the farmland birds that share their land” as part of its most recent Big Farmland Bird Count (GWCT 2022a, b). Others, however, are targeted more at knowledgeable naturalists, such as Inventera Åkerogräs (‘arable weed inventory’) in Sweden, which is coordinated by the Swedish Botanical Society, and whose participants often have little or no contact with the relevant farmer. Again, in all cases, volunteers’ involvement does not extend beyond the collection and submission of data.

Type 6: farmland-specific; fixed method and site selection This type consists of programmes that enable measurement of the effects of specific farmland habitats or farming practices on one or more aspects of biodiversity. Compared with those in Type 5, these programmes are characterised by a targeted selection of sites, either using a systematic grid as in Type 3 (but restricted to farmland), or sites with a specific type of management or farming intervention. These programmes are typically smaller-scale than those in Types 1 to 5, taking place across a limited number of carefully-selected sites—commonly fewer than 100, with a few small pilot programmes even consisting of ten sites or fewer. All such programmes are set up for the purposes of addressing a specific question, either as part of an academic study, or as an attempt by a conservation or agricultural NGO to evaluate the effects of a certain farming practice.

Programmes in Type 6 typically place considerable emphasis on volunteers either having existing skills, or receiving training specifically for the data collection to be carried out. There are, for example, references to “trained observers” (ARGOS Farmland Bird Monitoring Scheme, New Zealand), “experienced birdwatchers” (Birds on Farms, Australia) and those forming part of a University’s “Master Gardener” programme (Squash bee flower visitation study, USA). In around one third of the Type 6 programmes it is farmers who carry out data collection, and in most cases there is some reference to them either receiving training from the researchers carrying out the study, or detailed materials to aid with data collection methods and species identification. An exception is the BIMAG moth monitoring scheme on farmland in the Netherlands, where farmers operate moth traps, but identification skills are not required because species are identified by AI-based image recognition with validation by experts. One programme, Birds on Farms (Australia), is set up so that farmers accompany volunteers to carry out data collection, with a view to developing the necessary skills to continue it themselves. This greater emphasis on existing skills is likely due to the smaller scale of programmes of this type. That is, although the methods used are relatively simple, the small number of study sites means that there is less ‘room’ for observer error and a greater need for accuracy in the data collected.

Across all but one of these programmes, volunteers’ (including farmers’) involvement remains ‘contributory’. The one exception concerns the *development* of a monitoring programme in cooperation with farmers, which engaged them in workshops to help determine the key indicators to be monitored (Tasser et al. 2019). The smaller size of programmes of this type, however, arguably brings greater potential for farmers’ informal engagement with the research process. This may include granting volunteers access to their land, contact with researchers as they agree to be part of a programme, or face-to-face contact with volunteers as they make regular visits to the farm. In this type of programme, there is also more reference to farmers receiving feedback from volunteers or researchers on the results of the monitoring and what this means for their farming practices (e.g. RSPB Farmland Bird Surveys, UK).

**Type 6 examples: Squash bee flower visitation study (United States of America) and Taoyuan Farm Ponds Project bird survey (Taiwan)**

Appenfeller et al. (2020) detail a citizen science project developed specifically for their study, in which data collection was carried out by volunteers with existing specialist knowledge, on farms across Michigan. There is no mention of farmer involvement in data collection, although setting up the study is likely to have required some form of communication with farmers. The study focused on the squash bee—a specialist pollinator of pumpkins, squashes, and gourds, and therefore an important farmland bee species – and how its “flower visitation frequency varies according to crop management”. This was a relatively large study for its type, with 291 pollinator surveys carried out by 59 volunteers. The authors were thus able to draw strong links between specific agricultural practices and pollinator abundance.

To illustrate the variety within this programme type, we also include an example described by Chao et al. (2021) of a bird survey forming part of the Farm Ponds Project in the Taoyuan Tablelands of northwestern Taiwan. This example stood out in our review for being located outside of Europe and North America. Despite the relatively recent growth of citizen science, the project is also fairly long-established, running since 2003. Farm ponds, explain the authors, are “important for creating waterbird refuges to secure habitats for wintering waterbirds in anthropogenically influenced areas” (p.2). The bird surveys take place across 45 sites, and enable analysis of the effects of these particular habitats on bird populations. The authors do not directly specify the extent of farmers’ involvement in the surveys, but the 24 volunteers interviewed in their paper are all amateur naturalists, with the majority of these living in Taoyuan City.

Type 7: supporting small-scale farmer-led investigations Programme Types 7 and 8 represent a small number of programmes that are more notable for the ways in which they engage farmers than the data collection they enable. In Type 7 programmes, small groups of farmers are supported by an advisor or facilitator (often a researcher) to run on-farm experiments in which they have themselves identified the question to be addressed and the species or habitat to be monitored. We identified two examples of this programme type, both in the UK: Innovative Farmers, and Farmer Clusters. In some ways, these programmes represent an even more localised version of those in Type 6. They are, however, distinguished by their close involvement of farmers, and the relative lack of data from which knowledge can be applied elsewhere. It should also be noted that neither of the programmes identified is specifically focused on biodiversity, although each includes a number of “field labs” with a biodiversity-focused element.

In beginning with a question identified by a farmer or group of farmers, the experiments comprising these programmes are to a large extent ‘co-created’ as opposed to ‘contributory’ (Bonney et al. 2009) —that is, where non-professionals are involved in most or all stages of the research process, rather than simply contributing data to a programme designed and managed by others. Farmers play an active part in the design of these experiments, and in most cases carry out all data collection themselves. While the extent of farmers’ involvement in data analysis is unclear, they will at the very least be engaged with the results of the experiment and their implications, given that the experiment has stemmed from an issue or question they themselves have identified. It should be noted, however, that both identified programmes of this type are supported financially by national organisations who decide which applicants will receive funding, meaning that they are not entirely’bottom-up’ in their development.

**Type 7 example: Farmer Clusters (United Kingdom)** This programme aims to bring farmers together in “clusters” to carry out experiments, aided by an advisor or facilitator. Monitoring forms part of the programme, but is not linked to a particular study or wider dataset, with farmers instead setting up an experiment themselves (including applying for funding through the scheme), and potentially recruiting volunteers. Experiments (or “clusters”) within this programme therefore offer farmers a high degree of agency in terms of deciding on the overall purpose of the data collection, and what is to be monitored. The Farmer Clusters programme is ‘hosted’ by the Game and Wildlife Conservation Trust (GWCT), with a number of other organisations (mostly conservation-focused NGOs) acting as partners. The first clusters were established in 2015, and there are now roughly 120 of them across the UK.

Type 8: farmer engagement This final programme type comprises four programmes aimed solely at the engagement of farmers, rather than the collection of data for any other purpose. These consist of simple tools set up to encourage farmers to monitor biodiversity on their farm, purely for their own learning. These programmes can still be considered “citizen science” in that non-professionals are engaged in some form of standardised data collection, but the data collected are not currently used for research purposes and are secondary to their primary aim of farmer engagement. There is no required expertise or specified time commitment for farmers to participate in these programmes—monitoring can take place as little or as often as they choose, and the materials are intended primarily as engagement tools.

Type 8 example: LEAF Sustainable Biodiversity (United Kingdom) This is a booklet that forms part of the “Simply Sustainable” series of resources provided by the NGO Linking Environment and Farming (LEAF). The booklet aims to “help farmers monitor, manage and enhance biodiversity through the adoption of Integrated Farm Management (IFM)”, and details a series of simple steps to be taken towards this. Monitoring and identifying key species, on a site chosen by farmers themselves within their own farm, are the first two of these steps. Unlike Farmer Clusters and Innovative Farmers, there is no research question underlying this monitoring—the idea is simply to begin by identifying the biodiversity on their land (or a particular part of it). As far as can be determined by the programme’s website, the data then does not contribute to any wider dataset or scientific study.

## Discussion and conclusions

### Summary of review and typology

Through this review, we have identified 106 programmes employing citizen science approaches to farmland biodiversity monitoring. Based on these, we then created a typology of eight types that reflect considerable variation across these approaches. The distinctions used in the typology reflect the broad themes of data collection and volunteer engagement. In terms of the types of data collected, the key distinctions used in our typology among the first six programme types are: 1) the ‘general’ or ‘farmland-specific’ nature of a given programme; 2) the presence or otherwise of a specified data collection protocol; and 3) whether monitoring sites are chosen by volunteers or selected more systematically. Two further programme types then represent unique modes of farmer engagement.

Even though our search for citizen science programmes was not exhaustive, it is clear that certain types contain much larger numbers of programmes than others. We found particularly few programmes in Types 4, 7 and 8. This may be due in part to these types of programmes being less visible to our search methods, especially for the farmer-centric Types 7 and 8. For Type 4, i.e. farmland-specific schemes with free method and site selection, we believe that the low number of programmes is accurate, due to the combination of being a poor match to naturalist interests and an unclear offer to those outside the naturalist realm (including farmers—see for more below).

Our search strategy was designed to find farmland-specific monitoring programmes. It is therefore not surprising that most of the ‘general’ programmes (especially Types 1 and 2) were identified through partner contributions rather than through the literature and Google searches. That we found few such programmes through our (farmland-oriented) literature search, however, also indicates that the resultant data are rarely used to answer questions specifically about farmland biodiversity, and that their relevance to farmland-specific biodiversity monitoring might therefore be limited. It was also predictable that the most ‘standardised’ types of programmes, i.e. Types 3 and 6, were the ones most likely to be identified through the literature search. This is because the systematic nature of data collection makes these programmes particularly suited for empirical research leading to scientific publications.

In the following, we reflect on the current collective capacity of citizen science approaches to farmland biodiversity monitoring based on the programmes identified in this review, focusing on the two broad themes reflected in our typology: gathering data and engaging volunteers. As noted in the Introduction, both the data itself, and the engagement of new audiences in the process of gathering these data, can ultimately contribute to the conservation, restoration or enhancement of (farmland) biodiversity. We first discuss the strengths and limitations of the identified programmes in terms of the quantity and quality of the gathered data (summarised in Table 3), and reflect on these to inform future programme development. We then argue that any significant increase in the contribution made by citizen science to research on farmland biodiversity would require greater consideration of different groups of participants and their potential motivations to partake in, or even initiate, citizen science programmes. Finally, we discuss the various forms that this engagement could take. Possible ways forward, and their potential benefits for farmland biodiversity monitoring, are summarised in Table 4. It should again be noted that our aim in this paper was to conduct a wide-ranging review of the broad *types* of approaches, and what these can offer to farmland biodiversity monitoring, rather than carrying out any formal analysis of—for example—the quality of the data gathered through a given programme, or the outcomes of farmer engagement in terms of their attitudes towards biodiversity.

**Table 3:** Summary of the strengths and limitations of the eight citizen science (CS) programme types identified in this review, considering both data collection and volunteer (e.g. farmer, naturalist) engagement

Programme type	Strengths	Limitations
*General programmes*		
Type 1: general; free method and site selection	• Large number of potential participants • Large volumes of data • Easy for volunteers to get involved	• Variable and uncertain data quality • Uneven spatial coverage • Low level of volunteer commitment (i.e. easy for volunteers to drop out) • Might be challenging to filter out data from farmland
Type 2: general; fixed method, free site selection	• Large number of potential participants • Large volumes of data • Standardised methods	• Uneven spatial coverage • Might be difficult to estimate farmland-specific trends • Higher threshold for volunteers to get involved (requires certain skills)
Type 3: general; fixed method and site selection	• Fairly large number of potential participants • Large volumes of data • Standardised methods and high data quality • Possibility for long-term data series from known locations	• Might be difficult to estimate farmland-specific trends • Higher threshold for volunteers to get involved (requires certain skills)
*Farmland-specific programmes*		
Type 4: farmland-specific; free method and site selection	• Relatively large number of potential participants • Easy for volunteers to get involved • Farmland-specific data	• Variable and uncertain data quality • Uneven spatial coverage • Low level of volunteer commitment (i.e. easy for volunteers to drop out) • Apparently low numbers of participants and programmes
Type 5: farmland-specific; fixed method, free site selection	• Relatively large number of potential participants • Standardised methods • Farmland-specific data • May direct naturalists to record in farmland	• Uneven spatial coverage
Type 6: farmland-specific; fixed method and site selection	• Standardised methods and high data quality • Designed to answer questions about the effect of specific farming practices • May direct some naturalists to record on farmland • Can involve farmers specifically	• Relatively limited volumes of data • Time-limited • Time consuming to initiate (for the volume of data collected) • Ethical dimension of using CS for the collection of data typically collected by professionals
Type 7: supporting small-scale farmer-led investigations	• High level of volunteer engagement • Involves farmers specifically • Aims to answer questions that are relevant to farmers • High level of volunteer commitment	• Small volumes of data • Uncertain data quality • Small/local spatial coverage • Typically time-limited • Time consuming to initiate and run
Type 8: farmer engagement	• High level of volunteer engagement • Involves farmers specifically	• Gathering data for biodiversity monitoring is not the primary aim • Uncertain data quality

**Table 4:** Summary of recommendation for furthering citizen science approaches to farmland biodiversity monitoring, and their potential benefits

Potential action	Benefits to farmland biodiversity monitoring
*General recommendations*	
• Carefully consider whether a citizen science approach adds significant value to a given programme (e.g. in terms of desired data collected, engagement of farmers/volunteers) • Create opportunities for farmers and other members of agricultural communities to partake in farmland biodiversity monitoring based on their interests and potential motivations • Ensure timely, relevant and engaging feedback to participants	• Ensures effectiveness of programme from both a data collection and volunteer engagement perspective • Potentially considerable increase in data collection capacity; engagement of a (largely) new audience with the agency to adapt their land management practices based on the data collected • Prolonging engagement with the programme and enhancing opportunities for learning
*Recommendations for existing programmes*	
• Take measures to include more farmland/farmers within general monitoring programmes (Types 1 and 2)—e.g. developing project materials that encourage and enable farmers’ participation • Develop more farmland-specific low entry platforms that enable opportunistic submission of species observations (e.g. Type 4) • Adapt farmland-specific programmes employing free site selection (Type 5) to include more detailed information on monitoring sites (e.g. by asking farmers to submit details on habitat types and/or farming practices present)	• Considerable increase in data collection capacity informing farmland biodiversity trends over large spatial scales • Considerable increase in biodiversity trend data collection capacity at more local scales. • Addresses the spatial biases of general monitoring programmes employing free site selection, leading to greater farmland coverage; potentially enables more focused data that allows long-term monitoring of particular habitats or effects of farming practices on (aspects of) farmland biodiversity
*Recommendations for new programmes*	
• Develop further farmland-specific programmes employing fixed site selection and specific research questions (Type 6) • Co-create further programmes, involving farmers throughout the scientific process and working from their own interests (Type 7)	• Focused data on the effects of particular habitats or farming practices on (aspects of) farmland biodiversity, including monitoring to determine the effects of biodiversity-promoting measures • In-depth engagement of farmers with science and biodiversity monitoring, bridging of gaps between ‘communities of practice’

### Citizen science approaches to farmland biodiversity monitoring: current data collection capacity and future development

The varying nature of data collected through the different programme types make them suitable for responding to different questions or needs. The key strength of the large-scale non-farmland specific programmes in Types 1 and 2 (and to some extent in Type 3) is the collection of large volumes of data—submitted through user-friendly online platforms—that would be impossible without citizen science approaches (Chandler et al. 2017). Such data especially enable the analysis of general distribution trends of taxa including on farmland at national scales (Herzog and Franklin 2016). Type 1 programmes in particular lack a specified time commitment and do not demand high levels of species identification skills. However, such skills are central to the naturalist culture from which most biodiversity citizen science programmes develop (Ellis 2011; Sharma et al. 2019). It is not surprising, therefore, that several programmes develop or host (notably online) training tools or give feedback in various ways on submitted identifications, mechanisms which we know can increase both data quality and the volume of submissions (Van der Wal et al. 2016). Much of the data, however, may come from a smaller pool of very active volunteers with high skill levels (e.g. Kelling et al. 2015). The volume of data will then be determined by the popularity a species group (e.g. Knape et al. 2022), which thereby sets the opportunity provided by these general programmes to the monitoring of biodiversity trends on farmland at larger (e.g. national) spatial scales.

The opportunistic type of data from Type 1 (and its farmland-specific equivalent, Type 4) programmes has several challenges (Isaac et al. 2014; Rapacciuolo et al. 2021). One problem is that data from this type of programme typically consist only of presence records. Often, volunteers do not record all species they observe, instead recording only those that they find interesting, while ignoring the most common species (Isaac and Pocock 2015). This makes it impossible to know whether the absence of records means that certain species were indeed absent or simply not recorded. This issue can be mitigated by applying complete checklists, where observers report all the species they detect and identify. This makes it possible to separate non-detections from other reasons for not recording a given species, and can significantly improve the reliability of trends in species occupancy and distribution (Johnston et al. 2021).

Another problem with opportunistic data is uneven spatial coverage (Van der Wal et al. 2015; Mair and Ruete 2016). This occurs due to the tendency for recorders, when given free choice over their data collection site, to choose sites that –for example– are close to where they live (Isaac and Pocock 2015), or are known to have high species diversity (Johnston et al. 2023). This spatial bias can make it difficult to assess how general any observed trends are, including farmland-specific ones. Although Type 1 and 2 do not target farmland, the considerable size of some of those programmes means that coverage of such habitat can still be substantial. It may therefore be possible either to filter out data specifically from farmland sites, or to analyse trends for species that are known to occur mainly on farmland. Other challenges are that recording effort varies over time, across sites and between recorders, and that recorders might differ in their ability to detect or identify species of interest (Isaac et al. 2014). Methods for overcoming these issues include different types of data filtering, aggregating observations across space and time to reduce variability and uncertainty, estimating the likelihood of false absences through species co-occurrence patterns, accounting for sampling efforts in statistical models, and combining opportunistically and systematically collected data in the same models (Isaac et al. 2014; Rapacciuolo et al. 2021). Programmes in Type 2 have overcome the issues of uneven sampling effort, but the uneven coverage applies to this type as well. Nevertheless, the sheer amount of data generated through Type 1 and 2 programmes means that they can give very valuable information on large-scale biodiversity trends, including trends for species dependent on farmland.

The issue of spatial bias is partially offset in programme Type 3, where sites are allocated systematically to volunteers by a coordinating organisation. Many programmes in this category focused on birds use sites on a systematic grid, such that the sample is representative of different habitat types at a national scale. The analysis of large-scale temporal trends—for taxa that many volunteers are interested in—is made possible by the consistency of both monitoring sites and methods, as well as the long-running nature of programmes of this type—many date back to the late 1980s, some even to the 1960s, and all are ongoing rather than having a fixed end point. Indeed, data from this type of programme is used to calculate, for example, the Farmland Bird Index—a composite index founded on volunteer-based bird monitoring schemes across EU member states, used to assess the biodiversity status of agricultural landscapes in the EU (Rigal et al. 2023). Other examples include Young and Harrison's (2020) use of the Coordinated Avifaunal Road Counts scheme in South Africa to draw conclusions about one species—the blue crane—in agricultural areas. In regions where farmland is not a dominant land use and thus may end up being poorly represented in the total sample, it can however be difficult to use this type of data to estimate biodiversity trends for farmland specifically. Additionally, data collected through these programmes remain reflective of broad rather than habitat or farm-specific trends—for example, general trends for farmland birds at a national scale; they typically do not contain enough precision to connect these trends to specific farming practices or interventions (e.g. Calvi et al. 2018).

From a farmland biodiversity perspective, a key strength of programme Types 4 and 5 is their farmland-specific focus, which encourages data collection on sites where monitoring is unlikely to have previously taken place. The farmland-specific data gathered through these programmes also makes it easier to draw farmland-specific conclusions, compared to those of Types 1 and 2. Surprisingly, we only found one example programme of Type 4, which also appeared to have a relatively low number of participants. While this ought to be the type of farmland-specific citizen science with the lowest barrier to entry in terms of species identification skills and methodological constraints, we did not identify any such ‘mass farmland-specific programme’. This may indicate that it is difficult to attract volunteers to this type of programme. This might be because naturalists who participate in programmes in Type 1 (e.g. iNaturalist, eBird, Artportalen) are not attracted to farmland sites, and potential new volunteers from outside the naturalist community are more attracted to programmes with a standardised method where it may be clearer when a meaningful contribution (to e.g. science, contributing to greater knowledge on farmland biodiversity) is made. It is also possible that this type of programme is rare because it is unattractive to researchers, NGOs and other potential organisers of citizen science programmes who might be more interested in collecting standardised data. Across most Type 5 programmes, it is possible to account for farm and habitat types and farming practices or interventions. One example is Josefsson et al. (2017), who used data from the Swedish Volunteer and Farmer Alliance programme to draw links between agri-ecological conditions and bird abundance. The free site selection employed across these programmes, however, means that they share some of the limitations of Type 2, and suggests that they remain more suitable for assessing general farmland trends at a large scale than for any more focused analysis (Herzog and Franklin 2016).

In contrast to the data on overall trends that are the focus of programme Types 1 to 5, the key strength of programmes in Type 6 is that they enable analysis of the effects of specific farmland habitats or farming practices on specific aspects of biodiversity. Here, such focused analysis is made possible by the specific research questions asked and the subsequent careful selection of sites and participants. These programmes therefore appear to respond most clearly to the need for data enabling analysis of the effects of specific habitats, farming practices, or nature-enhancing measures on farmland biodiversity (Kleijn and Sutherland 2003). They do, however, still have important limitations from a data collection perspective compared to programmes in Types 1 to 5. While enabling more focused data analysis, the smaller size of these programmes and tendency to focus on one specific habitat type make them less suitable for investigating large-scale trends than those in Types 1 to 5. In contrast especially to programmes in Types 1 and 2, which yield amounts of data that would be virtually impossible without volunteers, the data in Type 6 programmes could in many cases be collected by professionals, although this would result in higher costs. One can, however, question whether it is ethical to engage volunteers if merely to reduce costs, particularly when a strongly structured (top-down) approach can regiment participants, thus limiting the influence of (for example) farmers on which biodiversity-related questions to address. It should also be noted that, especially for programmes of more limited size, it is probably safer to rely on professionals than on volunteers to ensure the long-term viability of the data collection. Additionally, the majority of programmes within this type are time-bound rather than ongoing, with many taking place over a few (typically three) seasons and stopping once their key questions have been addressed. In short, data collected through these programmes is typically well-suited to answering specific questions related to farming practices, but limited to particular habitats and short periods of time, while also raising ethical questions.

When using, or planning to use, citizen science as a way to monitor farmland biodiversity, it is important to be aware of the main strengths and limitations of the different types of programmes identified here, including those related to their respective primary audiences (Table 3). Different types of monitoring programmes are useful for different purposes and attract different participants. To summarise, given that farmland is represented to a high enough degree, non-specific large-scale programmes such as those in Types 1–3 can be used to estimate large-scale and long-term trends for species associated with farmland. Here, data from programmes with a standardised methodology and fixed sites are easier to use for estimating temporal trends, while data from Type 1 programmes might require several steps of filtering and more advances statistical techniques. Data from relatively large-scale but farmland-specific programmes (Type 5) are well-suited to estimating biodiversity trends for farmland specifically, but still often lack the information needed to relate these trends to farming practices. Type 4 programmes in particular, meanwhile, appear to lack appeal to volunteers and/or organisers. By contrast, more specified or local programmes—initiated either top-down (Type 6) or bottom-up (Type 7) with repercussions for participation and participants—can be designed to evaluate the effects of specific farming practices on biodiversity.

### Volunteer engagement: linking ‘communities of practice’

The need for data assessing the effects of measures to promote farmland biodiversity, combined with the spatial biases towards celebrated, biodiversity-rich and nearby places in ‘general’ citizen science programmes (Isaac and Pocock 2015; Mair and Ruete 2016), suggests that any significant increase in the contribution of citizen science to research on farmland biodiversity would either require engagement of more volunteers from rural areas, or making it more attractive for volunteers in general to monitor biodiversity on farmland. Our review points to two principal routes towards increasing the amount of volunteer-based biodiversity monitoring on farmland: 1) attracting existing (naturalist) volunteers to new sites, i.e. on farmland, and 2) engaging new groups of people who already spend a lot of time on farmland, for example farmers, in the monitoring. While our methodology did not allow for a quantitative assessment of the extent to which farmers are represented among naturalist volunteers, it was clear –from the thematic (qualitative) analysis of all identified programmes (Appendix S3) and in-depth knowledge among the author team of many of these programmes and their wider biodiversity monitoring context– that the level of engagement with farmers or other members of agricultural communities was low. Given their proximity to and daily engagement with their land, we find this remarkable. Additionally, in programmes where farmers *are* engaged, their involvement is limited to the collection and submission of data in a ‘contributory’ model of participation, as is the case for other volunteers. Notable exceptions here are the two programmes comprising Type 7, where experiments are devised by farmers themselves with the aid of an advisor, and farmers remain involved in all aspects of the scientific process thereafter. Furthermore, while many of the programmes in Types 4 and 5 target farmers specifically—albeit with varying success—their participation is likely to be limited in Types 1 to 3. Further research would be required, however, to test this prediction.

Building links between citizen science and agricultural communities might be characterised as bridging the gaps between two distinct ‘communities of practice’ (e.g. Oswald 2020; Sbrocchi et al. 2022). These are described by Sbrocchi et al. (2022, p. 2) as “typically informal, self-organising groups of individuals who advance their concerns or interests through regular interactions”. While bringing certain groups of people together through a common purpose or interest, such communities can also unwittingly create boundaries that make it challenging for those from outside that community to participate. Historically, biodiversity citizen science as a community of practice stems from amateur naturalists’ interests in species (Miller-Rushing 2012; Van der Wal et al. 2015) (although globally, there are notable examples demonstrating that large-scale programmes can stem from the interests or concerns such as those of local communities—e.g. Seagrass-Watch 2023). Owing to this historical context, and as shown by the spatial biases described in reference to programme Types 1 and 2, citizen science has tended to gravitate towards places other than farmland. As shown in this review, some programmes do attempt to draw farmers into this ‘community of practice’, but there is clear potential for finding more synergy by working from farmers’ interests and perspectives on biodiversity (e.g. Busse et al. 2021). Encouragingly, Garratt et al. (2019), reporting on farmers’ testing of pollinator and pollination service monitoring methods, found a general willingness and ability among farmers to carry out proposed survey methods, as well as (hypothetically) to implement them as a part of a wider citizen science programme. This willingness was partly due to the direct link between farm income and pollination services to crops, which suggests that connecting to farmers’ motivations could be a key way to encourage participation in such schemes.

As discussed in “Introduction” section, as well as adding significant capacity to farmland-focused data collection, there appears to be considerable potential for participation in citizen science to increase farmers’ interest in biodiversity and willingness to adopt more biodiversity-friendly farming practices, and even to form the basis for adaptive land management. That is, with farmers receiving direct feedback from their own data collection on their own land on the effects of land management practices or biodiversity-promoting interventions, farmers then have some degree of agency to adapt these practices (Billaud et al. 2021). Several papers identified in this review point to this potential through participation in citizen science (e.g. Tasser et al. 2019; Billaud et al. 2021), although it should be noted that further critical or empirical exploration of this potential has not been the purpose of this review, and further research is needed in order to provide this.

Within such future research, consideration should also be given to different forms and degrees of farmer engagement—and engagement of other agricultural community members—through citizen science. The programmes identified in this review provide examples of some of these. Here, across all programmes in Types 1 to 6, farmers’ only official involvement is in the collection and submission of data, suggesting that actual interaction with scientists, amateur naturalists or other members of the scientific community is limited. The focused monitoring programmes in Type 6 are likely to include higher levels of informal forms of engagement such as face-to-face contact with researchers or citizen scientists, or feedback from volunteers on the results of their monitoring, even when farmers do not carry out data collection themselves. It is the largely ‘co-created’ programmes comprising Type 7 that provide the most thorough or ‘in-depth’ engagement of farmers. Given that these programmes are driven by the direct interests of farmers, they also carry considerable potential in terms of identifying practices that require adaptive management. As noted previously, however, these programmes tend to consist of experiments carried out on a small number of sites, producing data that do not always contribute to any wider dataset or academic study. Additionally, the farmer-led nature of these programmes also represents a key difference from research driven by gaps in the scientific literature, or by a researcher’s own area of focus. While researchers can surely play a part in the further development and expansion of such programmes, this would most likely require a shift in perspective, prioritising the process of engagement over guaranteed research outputs (Beier et al. 2017). Such a shift could also make a long-term contribution to greater accessibility and “democratizing” of scientific research (Strasser et al. 2019, p. 66).

Finally, several researchers caution against the assumption that projects with higher levels of participation are necessarily ‘better’ than those where volunteers’ participation is limited to data collection and submission. Gunnell et al. (2021, p. 8) accept that “despite the reported benefits of a highly participatory approach”, such an approach may not always be necessary to achieve a project’s volunteer engagement aims (see also Haklay 2018). Elsewhere, Van der Gevel et al. (2020, p. 35) contend that “deep learning is possible in any project, as participants learn differently and engage with the project in unplanned ways”. These different forms and degrees of engagement, and how these may influence farmers’ attitudes towards biodiversity-friendly farming practices, should be a key area of focus within future research on this topic.

### Supplementary Information

Below is the link to the electronic supplementary material.

Supplementary file1 (PDF 461 KB)
