# Supplementary material for: Farmland biodiversity monitoring through citizen science: A review of existing approaches and future opportunities
Source: Ambio. 2023 Nov 16;53(2):257–75. doi: 10.1007/s13280-023-01929-x (PMC10774504; doi:10.1007/s13280-023-01929-x)
Supplement: Supplementary file 1 — Supplementary file1 (PDF 461 KB) [file 13280_2023_1929_MOESM1_ESM.pdf]

Ambio

Supplementary Information

This supplementary information has not been peer reviewed.

Title: **Farmland biodiversity monitoring through citizen science: A review of existing approaches and future opportunities**

Andy Ruck, René van der Wal, Amelia S. C. Hood, Alice L. Mauchline, Simon G. Potts, Michiel F. WallisDeVries, Erik Öckinger

Appendix S1: Web of Science and Google search terms

The search terms used in our search of the **Web of Science** Core Collection were as follows:

Title search:
TI=((farm* OR agri* OR agro*) AND ("citizen scien*"´)

Author keyword search:
AK=((farm* OR agri* OR agro*) AND ("citizen scien*"))

Wider author keyword search:
AK=((farm* OR agri* OR agro*) AND (biodivers* OR wildlife* OR conservation* OR species* OR taxa OR ecosystem* OR habitat* OR bee* OR pollinat* OR bird* OR beetle* OR vascular plant* OR butterfly* OR syrphid* OR hoverfly* OR earthworm*) AND ("citizen scien*" OR "public scien*" OR "civic scien*" OR "community scien*" OR participat* OR "public engag*" OR volunteer* OR non-professional* OR collectiv* OR farmer-led OR "farmer* led" OR monitor*))

In our **Google search**, we followed the first 100 results from each of the search terms below:

citizen science project farm biodiversity

farm* AND bird* AND count*

farm* AND bird* AND monitor*

farm* AND bird* AND survey*

farm* AND bird* AND count* OR monitor* OR survey*

farm* AND pollinat* OR bee* AND count* OR monitor* OR survey*

farm* AND flower* AND count* OR monitor* OR survey*

farmer AND scien* AND biodiversity AND partnership OR cooperat*

farmer AND biodiversity AND count* OR monitor* OR survey*

farmer AND scientist AND partnership AND biodiversity

farm* science* work together monitor*

farmer* AND bird* citizen scien*

farmer AND conservation AND count* OR monitor* OR survey*

farmer AND wildlife AND count* OR monitor* OR survey*

farm* AND butterfly* AND count* OR monitor* OR survey*

farm* AND insect* AND count* OR monitor* OR survey*

farm* AND beetle* AND count* OR monitor* OR survey*

farm* AND plant* AND count* OR monitor* OR survey*

farm* AND hoverfly* AND count* OR monitor* OR survey*

farm* AND worm* AND count* OR monitor* OR survey*

farm* AND earthworm* AND count* OR monitor* OR survey*

Appendix S2: Web of Science search full references

The following is a list of all 34 papers identified through our Web of Science search, and the programmes to which they refer.

Appenfeller, L.R., Lloyd, S. and Szendrei, Z., 2020. Citizen science improves our understanding of the impact of soil management on wild pollinator abundance in agroecosystems. *PloSa one*, *15*(3), p.e0230007. (Squash bee visitation monitoring programme, developed for this study)

Aue, B., Diekötter, T., Gottschalk, T.K., Wolters, V. and Hotes, S., 2014. How High Nature Value (HNV) farmland is related to bird diversity in agro-ecosystems–Towards a versatile tool for biodiversity monitoring and conservation planning. *Agriculture, ecosystems & environment*, *194*, pp.58-64. (German Common Breeding Bird Survey)

Billaud, O., Vermeersch, R.L. and Porcher, E., 2021. Citizen science involving farmers as a means to document temporal trends in farmland biodiversity and relate them to agricultural practices. Journal of Applied Ecology, 58(2), pp.261-273. (Farmland Biodiversity Obervatory/Observatoire Agricole de la Biodiversité, France)

Brereton, T., Roy, D.B., Middlebrook, I., Botham, M. and Warren, M., 2011. The development of butterfly indicators in the United Kingdom and assessments in 2010. *Journal of Insect Conservation*, *15*(1), pp.139-151. (UK Butterfly Monitoring Scheme)

Bretagnolle, V., Berthet, E., Gross, N., Gauffre, B., Plumejeaud, C., Houte, S., Badenhausser, I., Monceau, K., Allier, F., Monestiez, P. and Gaba, S., 2018. Description of long-term monitoring of farmland biodiversity in a LTSER. *Data in brief*, *19*, pp.1310-1313. (Zone Atelier Plaine et Val de Sevre, France)

Bretagnolle, V., Berthet, E., Gross, N., Gauffre, B., Plumejeaud, C., Houte, S., Badenhausser, I., Monceau, K., Allier, F., Monestiez, P. and Gaba, S., 2018. Towards sustainable and multifunctional agriculture in farmland landscapes: lessons from the integrative approach of a French LTSER platform. *Science of the Total Environment*, *627*, pp.822-834. (Zone Atelier Plaine et Val de Sevre, France)

Calvi, G., Campedelli, T., Florenzano, G.T. and Rossi, P., 2018. Evaluating the benefits of agri-environment schemes on farmland bird communities through a common species monitoring programme. A case study in northern Italy. Agricultural Systems, 160, pp.60-69. (Italian Common Bird Monitoring Programme)

Campedelli, T., Calvi, G., Rossi, P., Trisorio, A. and Florenzano, G.T., 2018. The role of biodiversity data in High Nature Value Farmland areas identification process: A case study in Mediterranean agrosystems. *Journal for nature conservation*, *46*, pp.66-78. (Italian Common Bird Monitoring Programme)

Chao, S.H., Jiang, J., Wei, K.C., Ng, E., Hsu, C.H., Chiang, Y.T. and Fang, W.T., 2021. Understanding pro-environmental behavior of citizen science: an exploratory study of the bird survey in taoyuan's farm ponds project. Sustainability, 13(9), p.5126. (Bird Survey forming part of Taoyuan Farm Ponds Project)

Damgaard, C., Moeslund, J.E. and Wind, P., 2020. Changes in the abundance of Danish orchids over the past 30 years. *Diversity*, *12*(6), p.244. (Monitoring of Danish Orchids)

Deschamps, S., & Demeulenaere, É. (2015). L'observatoire agricole de la biodiversité. Vers un ré-ancrage des pratiques dans leur milieu. Études Rurales, 195, 109–126. (Farmland Biodiversity Obervatory/Observatoire Agricole de la Biodiversite, France)

Gaget, E., Fay, R., Augiron, S., Villers, A. and Bretagnolle, V., 2019. Long-term decline despite conservation efforts questions Eurasian Stone-curlew population viability in intensive farmlands. *Ibis*, *161*(2), pp.359-371. (Zone Atelier Plaine et Val de Sevre, France)

Gerits, F., Messely, L., Reubens, B. and Verheyen, K., 2021. A social–ecological framework and toolbox to help strengthening functional agrobiodiversity-supported ecosystem services at the landscape scale. *Ambio*, *50*(2), pp.360-374. (1m2 gardens – part of landscape observatory BEL-lanscape, Flanders)

Gillings, S., Newson, S.E., Noble, D.G. and Vickery, J.A., 2005. Winter availability of cereal stubbles attracts declining farmland birds and positively influences breeding population trends. *Proceedings of the Royal Society B: Biological Sciences*, *272*(1564), pp.733-739. (BTO/JNCC UK winter farmland bird survey)

Herzog, F. and Franklin, J., 2016. State-of-the-art practices in farmland biodiversity monitoring for North America and Europe. Ambio, 45(8), pp.857-871. (Pan-European Common Bird Monitoring Scheme)

Hsu, C.H., Chou, J.Y. and Fang, W.T., 2019. Habitat selection of wintering birds in farm ponds in Taoyuan, Taiwan. *Animals*, *9*(3), p.113. (Bird Survey forming part of Taoyuan Farm Ponds Project)

Jerrentrup, J.S., Dauber, J., Strohbach, M.W., Mecke, S., Mitschke, A., Ludwig, J. and Klimek, S., 2017. Impact of recent changes in agricultural land use on farmland bird trends. *Agriculture, Ecosystems & Environment*, *239*, pp.334-341. (German Common Breeding Bird Survey)

Jiguet, F., Devictor, V., Julliard, R. and Couvet, D., 2012. French citizens monitoring ordinary birds provide tools for conservation and ecological sciences. *Acta Oecologica*, *44*, pp.58-66. (French Common Breeding Bird Survey)

Josefsson, J., Lokhorst, A.M., Pärt, T., Berg, Å. and Eggers, S., 2017. Effects of a coordinated farmland bird conservation project on farmers' intentions to implement nature conservation practices–Evidence from the Swedish Volunteer & Farmer Alliance. *Journal of Environmental Management*, *187*, pp.8-15. (Swedish Volunteer and Farmer Alliance)

Josefsson, J., Pärt, T., Berg, Å., Lokhorst, A.M. and Eggers, S., 2018. Landscape context and farm uptake limit effects of bird conservation in the Swedish Volunteer & Farmer Alliance. *Journal of Applied Ecology*, *55*(6), pp.2719-2730. (Swedish Volunteer and Farmer Alliance)

Jørgensen, P.S., Böhning-Gaese, K., Thorup, K., Tøttrup, A.P., Chylarecki, P., Jiguet, F., Lehikoinen, A., Noble, D.G., Reif, J., Schmid, H. and Van Turnhout, C., 2016. Continent-scale global change attribution in European birds-combining annual and decadal time scales. *Global Change Biology*, *22*(2), pp.530-543. (Pan-European Common Bird Monitoring Scheme)

Le Féon, V., Henry, M., Guilbaud, L., Coiffait-Gombault, C., Dufrêne, E., Kolodziejczyk, E., Kuhlmann, M., Requier, F. and Vaissière, B.E., 2016. An expert-assisted citizen science program involving agricultural high schools provides national patterns on bee species assemblages. Journal of Insect Conservation, 20(5), pp.905-918. (CS project in agricultural high schools, France)

Lee, M.S., Comas, J., Stefanescu, C. and Albajes, R., 2020. The Catalan butterfly monitoring scheme has the capacity to detect effects of modifying agricultural practices. *Ecosphere* 11(1), p.e03004. (Catalan Butterfly Monitoring Scheme)

MacLeod, C.J., Blackwell, G., Weller, F. and Moller, H., 2012. Designing a bird monitoring scheme for New Zealand's agricultural sectors. New Zealand Journal of Ecology, 36(3), pp.0-0. (ARGOS farmland bird monitoring scheme, New Zealand)

Nagy, S., Nagy, K. and Szép, T., 2009. Potential impact of EU accession on common farmland bird populations in Hungary. *Acta Ornithologica*, *44*(1), pp.37-44. (Hungarian Common Bird Monitoring Scheme)

Santangeli, A., Arroyo, B., Millon, A. and Bretagnolle, V., 2015. Identifying effective actions to guide volunteer-based and nationwide conservation efforts for a ground-nesting farmland bird. *Journal of Applied Ecology*, *52*(4), pp.1082-1091. (Enquête Busards-Milans/Montagu's Harrier, France)

Sardà-Palomera, F., Brotons, L., Villero, D., Sierdsema, H., Newson, S.E. and Jiguet, F., 2012. Mapping from heterogeneous biodiversity monitoring data sources. *Biodiversity and Conservation*, *21*(11), pp.2927-2948. (Ornitho, Catalunya)

Shaw, B.J. 2017. Citizen Science–Harnessing the Expertise of Farmers to Monitor Biodiversity in Austrian Meadows (Book chapter). (Biodiversitätsmonitoring mit LandwirtInnen, Austria)

Spasov, S., Hristov, J., Eaton, M. and Nikolov, S.C., 2017. Population trends of common birds in Bulgaria: Is their status improving after the EU accession. *Acta Zoologica Bulgaria*, *69*(1), pp.95-104. (National Bird Monitoring Scheme, Bulgaria)

Tasser, E., Rüdisser, J., Plaikner, M., Wezel, A., Stöckli, S., Vincent, A., Nitsch, H., Dubbert, M., Moos, V., Walde, J. and Bogner, D., 2019. A simple biodiversity assessment scheme supporting nature-friendly farm management. *Ecological Indicators*, *107*, p.105649. (Biodiversity assessment scheme developed for this study, in Alpine regions of France, Austria, Switzerland, Italy, Germany).

Weller, F., 2012. A comparison of different approaches to monitoring bird density on New Zealand sheep and beef farms. *New Zealand Journal of Ecology*, *36*(3), p.1. (ARGOS farmland bird monitoring scheme, New Zealand)

Wilson, S., Alavi, N., Pouliot, D. and Mitchell, G.W., 2020. Similarity between agricultural and natural land covers shapes how biodiversity responds to agricultural expansion at landscape scales. *Agriculture, Ecosystems & Environment*, *301*, p.107052. (North American Breeding Bird Monitoring Scheme)

Young, D.J. and Harrison, J.A., 2020. Trends in populations of Blue Crane Anthropoides paradiseus in agricultural landscapes of Western Cape, South Africa, as measured by road counts. *Ostrich*, *91*(2), pp.158-168. (Coordinated Avifaunal Roadcounts project, South Africa)

Zellweger-Fischer, J., Kéry, M. and Pasinelli, G., 2011. Population trends of brown hares in Switzerland: the role of land-use and ecological compensation areas. *Biological conservation*, *144*(5), pp.1364-1373. (Swiss Brown Hare Monitoring)

Appendix S3: Programme details

All programmes identified (through all three searches) were added to a spreadsheet, set up using Google Docs, that included a series of questions to be addressed for each programme, as detailed below. These questions, then, ran throughout all three searches used in this review. Co-authors were sent the questions when asked to add their contributions (see "Partner contributions" in section 2.3), as well as a document an information document that clearly explained each question.

- Name of programme.
- References to the programme (e.g. Academic articles, other).
- Website address (if available).
- What species/taxa are monitored?
- Scale of organization (e.g. national recording scheme, or locally-specific?)
- Who coordinates the programme? (e.g. Government, NGO, university, researchers)
- Who uses the data collected (if known)? (e.g. Government, NGO, university, researchers)
- Does the programme aim to monitor farmland specifically?
- Who does the monitoring? (e.g. farmers, citizen scientists, ecologists?)
- How do farmers/the agricultural community participate, if at all? (e.g. monitoring, receiving feedback from recorders, helping to identify the topic of study)
- If farmers are involved in the programme, who engages with them (e.g. ecologists, citizen scientists)?
- Does the programme aim to monitor: a) General biodiversity trends (e.g. of a certain species at national/local scale); b) The effects of something on biodiversity (e.g. an intervention, farming method, or policy); c) Both; d) Other (please specify)
- What types of methods are used? (And/or types of data collected?)
- Approximately how many sites are covered by the programme?
- How frequently is monitoring carried out?
- How long has the programme been running? (Or if no longer running, how long did it run for?)

- At what stage/s of the process are volunteers involved? (e.g. recording/submitting results, study design, data analysis):
- What does the data enable? (e.g. Identifying national trends, farm-level assessment of conservation measures)
- What are the strengths of the programme, to your knowledge/in your opinion? (e.g. data quantity/quality, farmer participation)
- What are the weaknesses of the programme, to your knowledge/in your opinion? (e.g. data quantity/quality, farmer participation)

Appendix S4: Table of programmes

<table>
<tr><th colspan="3">Type 1: General, free method and site selection</th></tr>
<tr><td>Country</td><td colspan="2">Summary</td></tr>
<tr><td>Sweden</td><td colspan="2">3 programmes: Artportalen (Species Portal); Naturens Kalender (Nature's Calendar); iGoTerra.</td></tr>
<tr><td>UK</td><td colspan="2">18 programmes: X:Polli-Nation – Photo submission; Mammal Mapper; National Moth Recording Scheme; Record Pool; UK Ladybird Survey; Soldierflies and Allies Recording Scheme; Nature's Calendar; iRecord; iSpot; Hedgehog Street; Ancient Tree Inventory; UK Beetle Recording Schemes; Bees, Wasps, & Ants Recording Schemes; British Bryological Society Recording Scheme; British Bugs Recording Schemes; British Leaf-miner Moths Recording Scheme; Lacewings & Allies Recording Scheme; British Myriapod & Isopod Group Recording Schemes; Society for the Study of Flies (Diptera) Recording Schemes.</td></tr>
<tr><td>Other</td><td colspan="2">5 programmes: eBird (USA); iNaturalist (worldwide); The Great Sunflower Project (USA); Ornitho (Cataluña, Spain); Irish Hedgehog Survey – opportunistic recording (Ireland).</td></tr>
<tr><th colspan="3">Type 2: General, fixed method, free site selection</th></tr>
<tr><td>Country</td><td colspan="2">Summary</td></tr>
<tr><td>Sweden</td><td colspan="2">4 programmes: Svensk Dagfjärilsövervakning (Swedish Butterfly Monitoring); Floraväktare (Flora Guardians); Rädda Bina (Save the Bees); Vinterfåglar Inpå Knuten (winter bird survey).</td></tr>
<tr><td>NL</td><td colspan="2">2 programmes: Argustelling (monitoring insects in Friesland); Dutch Bumblebee Monitoring Scheme.</td></tr>
<tr><td>UK</td><td colspan="2">11 programmes: UK Pollinator Monitoring Scheme - FIT counts; X:Polli-Nation – timed counts; UK Butterfly Monitoring Scheme; BeeWalk; Open Air Laboratories (OPAL) programme hedge-focused citizen science survey; National Bat Monitoring Programme - opportunistic surveys; Dragonfly and Damselfly Recording Schemes; Conker Tree Science Recording Scheme; National Plant Monitoring Scheme; Fungus Conservation Trust Recording Group.</td></tr>
<tr><td>Other</td><td colspan="2">Irish Hedgehog Survey - local area survey (Ireland).</td></tr>
<tr><th colspan="3">Type 3: General, fixed method and site selection</th></tr>
<tr><td>Country</td><td colspan="2">Summary</td></tr>
<tr><td>Sweden</td><td colspan="2">2 programmes: Svensk fågeltaxering (Swedish Bird Monitoring); Insect Biome Atlas.</td></tr>
<tr><td>NL</td><td colspan="2">3 programmes: Dutch Butterfly Monitoring Scheme; Dutch Macro-moth Monitoring Scheme; Dutch Dragonfly Monitoring Scheme.</td></tr>
<tr><td>UK</td><td colspan="2">4 programmes: BTO Breeding Bird Survey; Wider Countryside Butterfly Survey (Butterfly Conservation); UK Pollinator Monitoring Scheme – transect counts with pan trapping; National Bat Monitoring Programme - systematic surveys.</td></tr>
<tr><td>Other</td><td colspan="2">12 programmes: North American Breeding Bird Survey (USA/Canada/Mexico); Catalan Butterfly Monitoring Scheme (Cataluña, Spain); Coordinated Avifaunal Roadcounts project (South Africa); Monitoring of Danish Orchids (Denmark); Italian Common Breeding Bird monitoring programme (Italy); Hungarian Common Bird Monitoring Scheme (Hungary); National Common Bird Monitoring Scheme (Bulgaria); German Common Breeding Bird Survey (Germany); Suivi Temporal des Oiseaux Communs (France); Enquête Busards-Milans (France); Swiss Brown Hare Monitoring (Switzerland); Pan-European Common Bird Monitoring Scheme (various).</td></tr>
<tr><th colspan="3">Type 4: Farmland-specific, free method and site selection</th></tr>
<tr><td>Country</td><td>Programme name</td><td>Summary/Website or reference</td></tr>
</table>

UK	Rare Arable Flowers App	App created/run by the Biological Records Centre and supported by the Centre for Ecology and Hydrology. Specifically focused on wildflowers on arable land, and encourages users to submit any sightings. https://www.brc.ac.uk/app/rare-arable-flowers-app
**Type 5: Farmland-specific, fixed method, free site selection**		
**Country**	**Programme name**	**Summary/Website or references**
Sweden	Inventera Åkergräs: 'Free' inventories (opportunistic)	Monitoring of weeds on arable land. Consists of two methods, including 'free' inventories – i.e. volunteers choose a site to monitor, as opposed to being allocated a 'priority' site. https://svenskbotanik.se/inventera-akerogras/
Sweden	Lantbrukare och Fågelskådare (2007-10)	A pilot programme where bird habitats on farms (75 initially) were inventoried. Managed by the Swedish University of Agricultural Sciences (SLU), and based on the RSPB Volunteer and Farmer Alliance (UK, see below). The project ran until around 2010, but the SVFA programme (below) then stemmed from it. https://birdlife.se/projekt/tidigare-projekt/lantbrukare-och-fagelskadare/
Sweden	Swedish Volunteer and Farmer Alliance	SVFA "aimed to moderate negative farmland bird population trends, by promoting the implementation of conservation measures at the farm level". It included monitoring carried out by volunteers. https://birdlife.se/projekt/tidigare-projekt/lantbrukare-och-fagelskadare/
NL	Bescherming Boerenlandvogels	Farmers are trained to monitor and farm in a bird-friendly manner. Volunteers go out for at least half a day every week to monitor in a fixed setting – i.e. in the same place, on the same farm. https://www.boerenlandvogelsnederland.nl/
NL	Bond van Friese Vogelwachten	Volunteer conservation in Friesland initiative involving birdwatchers and including surveys. https://www.friesevogelwachten.nl/nl
UK	Big Farmland Bird Count	Similar to the many national bird surveys that make up programme Type 3, but with a specific focus on farmland and the engagement of farmers. https://www.bfbc.org.uk/
UK	RSPB Volunteer and Farmer Alliance (1999 - 2012)	A pre-cursor to the RSPB Farmland Bird Surveys (see below), where over 4,000 farms were surveyed by RSPB volunteers on opportunistically-selected sites.
UK	GWCT Partridge Count	Monitoring scheme of a typical farmland bird, mostly carried out by farmers. https://www.gwct.org.uk/partridge
UK	BTO/JNCC winter farmland bird survey (1999-2003)	Four-year survey of birds on farmland in winter, consisting of 1km square counts carried out by "volunteer surveyors". https://www.bto.org/our-science/publications/research-reports/winter-farmland-bird-survey
UK	On-farm earthworm survey	Pilot study which mobilised farmers to assess over 1300 ha farmland soils in spring 2018. https://www.globalsoilbiodiversity.org/blog-beneath-our-feet/2019/3/22/4ewwexsdm77v9qxyrp1hswhzvg605h
UK	Open Farm Sunday Pollinator Survey	One-off count in which volunteers were invited to farms to carry out pollinator surveys (2012). http://cehsciencenews.blogspot.com/2012/12/citizen-scientists-ensure-success-of.html

France	Observatoire Agricole de la Biodiversité	The Farmland Biodiversity Obervatory "offer(s) protocols for observing ordinary biodiversity to interested farmers, with a view to better understanding ordinary biodiversity in an agricultural environment and its links with practices" (Billaud et al. 2021). https://www.observatoire-agricole-biodiversite.fr/
France	Citizen science programme with agricultural high schools	"An expert-assisted citizen science program where teachers from 20 French agricultural high schools collected bees, which were identified to species level by a panel of expert bee taxonomists." (Le Feon et al. 2016).
Spain	Observatorio de Biodiversidad Agraria	A commitment to creating a monitoring network of agricultural biodiversity that can evaluate the impacts of agricultural management, involving farmers in data collection. Currently small-scale but with plans to expand. https://oba.fundacionglobalnature.org/
Austria	Biodiversitätsmonitoring mit Landwirt-Innen	"A nationwide network of more than 700 Austrian farmers and 12 agricultural schools regularly observe rare plants and animals in their own species-rich grasslands" (Shaw 2017).
USA	Songbird Farm Trail	Mostly a conservation scheme encouraging farmers to install nest boxes, but also includes monitoring. https://www.wildfarmalliance.org/songbird_farm_trail
Canada	GrassLander	Small scheme set up by a professor and Masters student, surveying eastern meadowlark and bobolink on farmland. One of the aims is encouraging farmers to delay haying in order to improve the survival chances of the birds. https://www.wlu.ca/news/spotlights/2017/july/laurier-based-citizen-science-project-tracking-threatened-birds-on-farmland.html
**Type 6: Farmland-specific, fixed method and site selection**		
**Country**	**Programme name**	**Summary/Website or references**
Sweden	Inventera Åkerogräs: Prioritised sites (systematic)	Monitoring of weeds on arable land. Consists of two methods, including this scheme, where volunteers are allocated a 'priority' site to monitor by the coordinating organisation. https://svenskbotanik.se/inventera-akerogras/
NL	Meadow bird agreement with agri-environment cooperatives	"The results-based element of the scheme requires participants to map and monitor meadow bird nests on their land as a proxy for the number of breeding meadow birds on site.... Farmers are responsible ultimately for counting and monitoring of the meadow bird nests in conjunction with local conservation volunteers". https://ec.europa.eu/environment/nature/rbaps/fiche/meadow-bird-agreement-agri-environment-cooperative_en.htm
NL	Dutch Agricultural Scheme for Dragonflies (Agrarisch meetnet libellen)	AML involves about 25 volunteers and professionals counting the dragonfly green hawker (*Aeshna viridis*) along a fixed route on the banks of ditches. The project operates across four provinces, and aims to compare the effect of traditional land-use practices with nature friendly agricultural schemes. https://www.vlinderstichting.nl/agrarisch-meetnet-libellen/
NL	Pollinator Monitoring of Bird-friendly cropland	Bird-friendly cropland consists of lucerne or red clover crop strips alternating with strips of herb-rich mixtures to benefit farmland birds. Pollinator monitoring was conducted to evaluate the value of this practice for bees and hoverflies.

		https://www.vlinderstichting.nl/wilde-bijen-in-groninger-vogelakkers
NL	Farmer Insect Monitoring on Agricultural Land (BIMAG)	In order to involve farmers in enhancing insect diversity, this monitoring project targeting macro-moths and butterflies was initiated as a collaborative project between farmer organisations and Dutch Butterfly Conservation. https://www.vlinderstichting.nl/bimag/
NL	ANLb Policy Monitoring for Amphibians and Fish	This programme aims to support provinces and agri-environment associations in monitoring and evaluating the effectiveness of agri-environment policy. https://www.ndff.nl/overdendff/validatie/protocollen/1-204-anlb-meetnet-amfibieen-en-vissen/
UK	RSPB Farmland Bird Surveys	Farmers can choose to be part of this project, in which "volunteers undertake surveys to monitor population trends of priority bird species, and we provide targeted advice to ensure appropriate conservation management". Includes more specific programmes within this overarching scheme (e.g. Strathspey Wetland and Wader Initiative). https://www.rspb.org.uk/our-work/conservation/conservation-and-sustainability/farming/advice/get-a-farmland-bird-survey/
UK	English Farm Woodland Bird Monitoring Scheme	Focused, time-bound scheme in 2019, assessing how well birds have colonised new "farm woods" planted since 1999. https://www.bto.org/our-science/projects/english-farm-woodland-bird-survey-0
UK	Northumberland Coast farmland bird monitoring	Small farmland bird monitoring scheme, starting by pairing 10 volunteers with 10 farmers. Specifically developed for mixed farming system there, which is not typical of UK farming. https://www.northumberlandcoastaonb.org/farmland-bird-monitoring/
UK	Short-haired Bumblebee Reintroduction Project	Project aimed at re-introducing the short-haired bumblebee. Working with farmers, conservation groups and smallholders in a particular area - Dungeness and Romney Marsh in Kent/East Sussex. Includes monitoring carried out by "trained volunteers". https://www.bumblebeeconservation.org/short-haired-bumblebee-reintroduction-project/
France	Projects forming part of Zone Atelier Plaine & Val de Sèvre (ZA PVS)	An LTSER (long-term social and ecological research) project in an experimental agricultural area that involves collaborative research with farmers. (Bretagnolle et al. 2018).
Alps region (5 coun-tries)	Development of Biodiversity assessment scheme	Biodiversity assessment scheme developed with farmers for the sake of this study. Including workshops to agree upon the indicators, then research carried out (with 13 out of 44 farmers participating) (Tasser et al. 2019).
Belgium	1m2 gardens (part of landscape observatory BEL-landscape)	Scheme in which volunteers maintain a 1-metre-squared area of land to monitor ecosystem services provided by different crops. Not 100% about biodiversity but the project as a whole is about "functional agricultural biodversity (FAB)". https://www.bel-landschap.be/
Austra-lia	EnviroDNA	Citizen science project investigating farm dam biodiversity in West Gippsland, Victoria: Landholders and other Landcare locals went in search of wildlife DNA, using innovative technology called environmental DNA (eDNA). https://www.youtube.com/watch?v=494Q2_34WK8&t=45s

Austra-lia	Birds on Farms	CS project, in Victoria but expanding into NSW and elsewhere, where volunteers count birds on privately-owned land (including farms). "Sometimes these surveys are undertaken in conjunction with the landholder as part of an informal training session." https://birdlife.org.au/projects/woodland-birds-for-biodiversity/birds-on-farms-wl
New Zealand	ARGOS farmland bird monitoring scheme	Monitoring scheme that aims "initially to establish baseline information on community composition and species distribution and abundance in relation to different farming systems and locations" (MacLeod et al. 2012).
USA	Squash bee flower visitation study	Developed for a study in Michigan where citizen scientists collected and submitted data on an important farmland wild bee species – the squash bee. (Appenfeller et al. 2020).
Taiwan	Bird Survey forming part of Taoyuan Farm Ponds Project	"The Bird Survey in Taoyuan's Farm Ponds Project is a systematic citizen science project founded in 2003. This project aims to identify existing and potential irrigation ponds that are important for creating waterbird refuges" (Chao et al. 2021).
**Type 7: Supporting local farmer-led investigations**		
**Country**	**Programme name**	**Summary/Website or references**
UK	Innovative farmers	"Innovative Farmers is a network of farmers and growers who are running on-farm trials, on their own terms". https://www.innovativefarmers.org/
UK	Farmer Clusters	Brings farmers together in clusters to "work more cohesively together in their locality" on studies of their own design, aided by an advisor or facilitator. https://www.farmerclusters.com/advice/monitoring-species/farmer-cluster-wildlife-surveys/
**Type 8: Farmer engagement**		
**Country**	**Programme name**	**Summary/Website or references**
Ger-many	MonViA monitoring tool (under develop-ment)	"MonViA's development and implementation of a Citizen Science-based Monitoring tool aims to facilitate the voluntary participation of farmers…. to document biodiversity indicators on farms, to be mindful of nature and actively promote biodiversity through agriculture". https://enrd.ec.europa.eu/evaluation/knowledge-bank/citizen-science-based-monitoring-agricultural-biodiversity-tool_en
UK	Cool Farm Tool	A tool for farmers to monitor greenhouse gases, water (quality), and importantly, biodiversity. https://coolfarmtool.org/coolfarmtool/biodiversity/
UK	LEAF simply sustainable biodiversity	A booklet to help farmers monitor, manage and enhance biodiversity "through the adoption of Integrated Farm Management (IFM)." https://leaf.eco/farming/simply-sustainable-series
UK	FWAG farmland monitoring tools	Like the above two, appears to be a monitoring tool for farmers, but just to help them see the benefits - not part of a citizen science scheme. http://adlib.everysite.co.uk/adlib/defra/content.aspx?id=000HK277ZW.09SIPKQGA1M4TJ
